# Nurses’ knowledge, attitude, and fall prevention practices at south Korean hospitals: a cross-sectional survey

**DOI:** 10.1186/s12912-020-00507-w

**Published:** 2020-11-24

**Authors:** Mi-young Cho, Sun Joo Jang

**Affiliations:** 1 Graduate School of Advanced Nursing Practice, Eulji University, Daejeon, Republic of Korea; 2 Red Cross College of Nursing, Chung-Ang University, 84 Heukseok-ro, Dongjak-gu, Seoul, 06974 Republic of Korea

**Keywords:** Attitude, Falls, Hospital, Knowledge, Nursing

## Abstract

**Background:**

Fall-prevention activities are nursing interventions which are designed to improve patient safety. The introduction of evaluations of medical institutions and an increase in medical litigation has led institutions to emphasize the importance of fall-prevention activities. The current situation regarding falls among patients in small and medium-sized hospitals is poorly understood. This study assessed knowledge and attitudes regarding falls, and fall-prevention activities of nurses working in small- and medium-sized hospitals.

**Methods:**

Nurses (*N* = 162) from seven small- and medium-sized hospitals participated in the study. Data on participants’ characteristics, education regarding patient falls, knowledge of stretcher cart use, attitudes regarding patient falls, and fall-prevention activities were collected from August 1 to September 1, 2016.

**Results:**

Nurses’ knowledge of patient falls was positively correlated with their experience with inpatient falls. Furthermore, nurses’ attitudes regarding falls were influenced by their nursing experience and fall prevention education. Attitudes positively correlated with fall-prevention activities, but knowledge did not. Nurses’ attitudes regarding patient falls were correlated with fall-prevention activities.

**Conclusion:**

Hospitals should develop incentive programs to improve nurses’ attitudes which are based on their subjective norms and tailored to each hospital’s specific circumstances to ensure engagement in fall prevention activities. In short, we recommend that consistent, repeated, and custom fall-prevention education should be implemented in small- and medium-sized hospitals to promote engagement in fall-prevention activities. Patient safety activities in small- and medium-sized hospitals can be enhanced by creating an environment that encourages active and self-directed participation in developing fall-prevention strategies using motivation and rewards.

## Background

Falls in hospitals are the most frequently reported incidents among all safety accidents and can lead to significant complications in patients [1–3]. Falls are considered a serious concern because they lead to financial losses and patient discomfort and affect patients’ quality of life by prolonging hospitalization and incurring additional medical expenses for tests, surgery, or rehabilitation [4, 5].

According to the Korea Consumer Agency’s annual report on patient safety, falls in hospitals accounted for 1522 (49.7%) of 3060 cases related to hospital safety management and constituted the largest proportion of safety accidents [6]. Fall rates for hospital inpatients vary considerably according to each medical institution’s incident report regulations and standards [7], and the number of reported cases is lower relative to that of actual occurrences [8]. Furthermore, determining accurate fall rates for all domestic hospitals is difficult, because hospitals do not release fall reports. Patients have the right to safety and quality management to prevent falls and injuries [3, 9], especially because falls are preventable and predictable health issues which can generally be reduced 30–50% with environmental improvements [10]. In addition, some researchers have suggested that inpatient falls can indicate nursing quality [11]. For these reasons, it behooves us to better understand falls and fall-prevention practices for the well-being of patients, nurses, and hospitals alike.

Small- and medium-sized hospitals improve accessibility to healthcare, increase the speed of providing emergency care, and serve as the backbone of the medical delivery system by providing patients with appropriate and diverse services for their medical needs [12]. A total of 89.2% of all hospitals in South Korea in 2013 were small- and medium-sized hospitals [13]. Although they accounted for two thirds of all medical institutions, the numbers of small- and medium-sized hospitals with patient safety departments, patient safety education, and patient safety indicators were lower relative to those of medical institutions with more than 300 beds [14]. In addition, a study that compared patient safety nursing activities between university hospitals and small- and medium-sized hospitals showed that the former performed more patient safety nursing activities pertaining to falls than the latter [15]. However, comparisons are difficult – research on the perception of hospital safety culture in South Korea has been conducted primarily in tertiary care medical institutions or medical institutions with more than 300 beds, and subarea classification criteria and analysis methods vary between studies [16]. The composition or characteristics of medical staff, hospital systems, patients’ illness, patients’ socio-demographic factors, and patients’ caregivers could differ between small- and medium-sized hospitals and large hospitals [17].

Falls in small- and medium-sized hospitals are not well understood because previous studies have been conducted mostly in tertiary care hospitals and few of these have involved small- and medium-sized hospitals [18, 19]. A previous study explored the knowledge of inpatient falls, attitude towards patient falls, and fall prevention activities among nurses in hospitals with more than 150 beds [20], but it was conducted only in geriatric settings. Moreover, small- and medium-sized hospitals encounter challenges such as a low perception of patient safety culture with regard to implementing effective strategies and practices to reduce patient falls [17, 21]. Hospitals with a high perception of patient safety culture had a lower rate of patient falls than hospitals that did not. Therefore, attempts to increase the perception of a safety culture have emerged as an important issue to reduce accidents [22, 23]. However, the perception of patient safety culture among nurses in small- and medium-sized hospitals was low, indicating that there is a difference in the perception of patient safety cultures according to the size of the hospital [21, 22]. According to the Agency for Healthcare Research and Quality [24], successful practice strategies begin with an understanding of current practice. Therefore, an understanding of the knowledge and attitudes of nurses working at small- and medium-sized hospitals, which affects engagement in fall-prevention activities, should be acquired to ensure better fall-prevention practices.

The present study examined nurses working in small- and medium-sized hospitals’ fall-related education and their knowledge, attitudes, and engagement in fall-prevention activities. We also explored differences in these factors and the relationships between nurses’ knowledge, attitudes, and engagement in fall-prevention activities.

## Methods

### Design

We conducted a cross-sectional study with surveying nurses in small- and medium-sized hospitals.

### Participants

Our participants were nurses who worked at small- and medium-sized hospitals with fewer than 300 beds located in D and G cities, who understood the purpose of the study, and who agreed to participate in the study. After obtaining permission from the institutes to conduct the survey, questionnaires were distributed by the respective nursing managers. Nurses working at Chinese medicine clinics and long-term care hospitals were excluded from the study.

The sample size necessary for correlation analysis with an effect size of .35, a significance level of *p* < .05, and power of .80 was 142, calculated based on a previous study [20] using G*power 3.1.9 [22]. Therefore, expecting a 20% attrition rate, we chose to include 190 nurses.

### Instruments

We used a previous study’s [20] questionnaires to determine participants’ demographic characteristics, in-patient fall experience, and fall prevention education.

#### Knowledge regarding falls

We assessed nurses’ knowledge regarding falls using the Hospital Falls Knowledge Scale, which measures nurses’ knowledge regarding falls [25] and was created in consultation with two nursing professors and two patient safety experts. The questionnaire consisted of 15 items pertaining to fall rates; types and severity of injuries caused by falls; intrinsic factors including high risk of falls, age group, sex, disease, state of consciousness, activity status, use of assistive devices, risk factors, and use of drugs that increased the risk of falls; and extrinsic factors including fall timing, location, and type. The questionnaire included 14 multiple-choice items and one subjective item (Item 15), and the highest possible score was 14. One point and zero points were awarded for correct and incorrect responses, respectively. The Kuder-Richardson Formula 20 reliability coefficient was .76. The content validity index for the modified instrument exceeded .80 for all items.

#### Attitudes regarding falls

Attitudes regarding falls refers to nurses’ emotional, cognitive, and behavioral reactions to inpatient falls that occurred in hospitals. We assessed these attitudes using the 13-item Hospital Falls Attitude Scale [26], which was used in a previous study [27]. Responses were provided using a five-point Likert scale ranging from 1 (*strongly disagree*) to 5 (*strongly agree*). Higher scores indicated higher levels of positivity in nurses’ attitudes. Items 2, 8, 9, 11, and 12 were reverse scored. Cronbach’s αs for the scale were .75 in a previous study and .72 in the present study.

#### Fall-prevention activities

Nurses’ engagement in fall-prevention activities was measured using a 20-item scale [18], which was based on the Hospital Nurses Association’s nursing practice guidelines for falls. Responses were provided using a four-point Likert scale ranging from 1 (*never use*) to 4 (*always use*), and higher scores indicated greater engagement in fall-prevention activities. Cronbach’s a for the scale was .92 in a previous study as well as in the present study.

### Data collection

Data were collected from nurses working at small- and medium-sized hospitals with fewer than 300 beds located in D and G cities from August 1 to September 1, 2016. The 190 nurses eligible were given explanations regarding the purpose and methods of the research and questionnaires were distributed among those who wanted to participate with an enclosed envelope for return. The envelopes were sealed and submitted after completion.

### Ethical considerations

Ethical approval for the study was granted by the institutional review board of Eulji University (approval number: IRB No. EU16–28), and the study protocol conforms to the provisions of the Declaration of Helsinki. Written informed consent was obtained from all participants.

### Data analysis

Participants’ characteristics and fall-related education were analyzed using frequencies, percentages, means, and standard deviations. Means and standard deviations were calculated for knowledge, attitudes, and engagement in fall-prevention activities. Differences in participants’ characteristics, knowledge, and attitudes regarding falls, and engagement in fall-prevention activities according to their experience of fall-related education were analyzed using Mann-Whitney U tests and Kruskal-Wallis tests. Pearson’s correlation coefficients were calculated for the relationships between knowledge regarding falls, attitudes regarding falls, and engagement in fall-prevention activities. Missing data were handled using listwise deletion. All data analyses were performed using SPSS/WIN 24.0.

## Results

Among the 190 distributed questionnaires, 4 were not returned, and 24 were blank. Therefore, the final analytic sample included 162 questionnaires (85.3% response rate). The participants’ mean age was 32.49 years (SD 8.17) and 157 (96.9%) of them were female. The mean duration of participants’ work experience was 8 years and 5 months. Of the total number of participants in the study, 82 (50.6%) nurses had experienced inpatient falls and 127 (78.4%) nurses had participated in educational programs on fall prevention.

### Differences in knowledge, attitudes, and engagement in fall-prevention by participants’ experience

Nurses’ experience with patient falls differed significantly depending on their experience. Nurses who had experience with patient falls had significantly higher levels of knowledge regarding falls than those who had no such experience. In addition, attitudes regarding falls differed significantly depending on how long our participants had been working as nurses. Nurses with 5 or more years of experience demonstrated significantly more positive attitudes toward falls than nurses with less experience. However, engagement in fall-prevention activities did not significantly differ by participants’ experience (Table 1).

**Table 1:** Differences in knowledge, attitudes, and engagement in fall-prevention activities according to participants’ characteristics (*N* = 162)

Characteristics	*n*	Knowledge				Attitude				Fall-prevention activities			
		*M*	*SD*	χ^*2*^	*p*	*M*	*SD*	χ^*2*^	*p*	*M*	*SD*	χ^2^ (*p*)	*p*
Age													
< 30 years	70	6.96	1.48	0.72	.488	3.78	0.38	1.04	.356	3.27	0.48	0.52	.597
30–39 years	59	6.64	1.74			3.80	0.32			3.27	0.50		
≥ 40 years	33	6.94	1.43			3.88	0.30			3.37	0.52		
Sex													
Male	5	7.20	0.84	0.56^a^	.578	3.75	0.15	0.64^a^	.525	3.21	0.45	0.49^a^	.628
Female	157	6.83	1.59			3.81	0.35			3.29	0.50		
Educational level													
Community college	81	6.79	1.73	0.76	.685	3.82	0.35	0.93	.630	3.30	0.49	0.68	.713
University	77	6.91	1.43			3.80	0.34			3.28	0.51		
Master’s degree or higher	4		0.58			3.69	0.32			3.13	0.49		
Work experience													
< 5 years	62	6.92	1.54	0.51^a^	.611	3.73	0.40	2.25^a^	.026	3.25	0.51	0.75^a^	.455
≥ 5 years	100	6.79	1.59			3.85	0.29			3.31	30.49		
Department													
Internal medicine	20	7.05	1.54	6.78	.079	3.82	0.24	0.85	.838	3.38	0.51	1.54	.674
Surgery	101	7.00	1.61			3.79	0.39			3.25	0.50		
Intensive care unit	22	6.32	1.13			3.85	0.25			3.34	0.50		
Other	14	6.21	1.58			3.82	0.29			3.31	0.52		
Experience of patient falling													
No	80	6.54	1.60	2.46^a^	.015	3.76	0.32	1.61^a^	.110	3.26	0.51	0.82^a^	.415
Yes	82	7.13	1.49			3.85	0.37			3.32	0.49		
Number of patient falls experienced													
1	32	7.25	1.68	5.89	.207	3.82	0.33	0.89	.926	3.30	0.54	1.21	.877
2	22	6.55	1.10			3.82	0.40			3.39	0.48		
3	14	7.43	1.55			3.90	0.44			3.34	0.44		
4	5	7.80	1.48			3.94	0.22			3.11	0.61		
≥ 5	9	7.33	1.32			3.91	0.41			3.30	0.39		
Fall-prevention education													
No	35	6.86	1.59	0.08^a^	.940	3.73	0.26	1.56^a^	.122	3.14	0.57	1.95^a^	.053
Yes	127	6.83	1.57			3.83	0.36			3.33	0.47		

*M =* mean; *SD =* standard deviation

^a^Mann-Whitney U-test result; *SD* Standard deviation

Nurses’ knowledge did not differ significantly by experience with fall-related education. Nurses’ attitudes regarding falls differed significantly according to the number of fall-prevention education sessions they attended. Nurses who had attended at least five fall-prevention education sessions demonstrated significantly more positive attitudes toward patient falls than nurses who had attended only one or two sessions. However, engagement in fall-prevention activities did not differ significantly by experience with fall-related education (Table 2).

**Table 2:** Differences in knowledge, attitude, and engagement in fall-prevention activities according to experience of fall-related education (*N* = 127)

Characteristic	*n*	Knowledge				Attitude				Fall-prevention activities			
		*M*	*SD*	*χ*^*2*^	*p*	*M*	*SD*	*χ*^*2*^	*p*	*M*	*SD*	*χ*^*2*^ *(p)*	*p*
Provider													
Nursing association	16	7.19	1.60	2.61	.456	3.89	0.32	6.00	.112	3.41	0.39	2.99	.394
Nursing society	3	6.33	1.15			4.18	0.25			3.43	0.55		
Hospital	106	6.77	1.58			3.82	0.37			3.32	0.48		
Other	2	8.00	1.41			3.46	0.44			2.90	0.14		
Instructor													
Nursing professor	16	7.19	1.33	3.40	.334	3.87	0.40	1.50	.683	3.26	0.52	3.34	.342
Nurse	107	6.79	1.61			3.83	0.36			3.35	0.47		
Hospital administrator	2	5.50	0.71			3.73	0.27			2.88	0.04		
Other	2	7.50	0.71			3.62	0.22			3.25	0.35		
Education method													
Lecture	38	6.66	1.55	2.82	.588	3.84	0.35	1.93	.749	3.31	0.57	1.78	.776
Audiovisual	5	7.2	1.30			3.69	0.25			3.20	0.27		
Lecture + audiovisual	79	6.91	1.63			3.82	0.37			3.35	0.44		
Lecture + discussion	3	6.00	1.00			3.92	0.20			3.17	0.47		
Other	2	7.5	0.71			4.15	0.76			3.53	0.25		
Education duration													
< 1 h	41	6.83	1.82	5.68	.224	3.79	0.41	6.15	.188	3.31	0.54	5.59	.232
1–2 h	60	6.93	1.38			3.82	0.36			3.28	0.45		
2–4 h	16	6.38	1.67			3.84	0.27			3.55	0.38		
4–6 h	3	8.67	1.53			3.92	0.08			3.53	0.48		
> 6 h	7	6.29	0.76			4.12	0.27			3.27	0.41		
Number of education sessions													
1	39	6.82	1.41	0.58	.966	3.74	0.39	22.68	<.001	3.21	0.52	3.79	.435
2	38	6.92	1.73			3.72	0.28	above 5 > 1,2^a^		3.37	0.44		
3	22	6.86	2.01			3.86	0.31			3.37	0.49		
4	6	6.67	1.03			4.06	0.31			3.55	0.39		
Above 5	22	6.73	1.24			4.08	0.37			3.36	0.42		

^a^Bonferroni correction; *M =* mean; *SD =* standard deviation

### Knowledge and attitudes regarding falls and engagement in fall-prevention activities

The mean proportion of correct answers on the items regarding knowledge about falls was 48.9% (an average of 6.84 out of 14 items). The proportion of correct answers for the items ranged from 3.7 to 95.1%. Item 5 (state of consciousness) had the lowest proportion of correct answers, and Item 2 (high-risk age) the highest.

The mean rate of positive attitudes regarding falls was 76.2% (Mean 3.81 out of 5, SD = 0.34; Table 3). The mean rate of engagement in fall-prevention activities was 82.3% (Mean 3.29 out of 4, *SD =* 0.50; Table 4).

**Table 3:** Average scores for attitudes regarding falls (*N* = 162)

Rank	Item	Items pertaining to attitudes regarding falls	*M*	*SD*
1	10	I think I should respond promptly when patients ask for help to move.	4.36	0.58
2	7	I think that patients’ fall risk should be assessed upon admission.	4.21	0.59
3	11	I do not think that fall-related physical injury is severe.^a^	4.20	0.73
4	6	I think I should actively nurse patients to prevent falls.	4.14	0.62
5	1	I am interested in the occurrence of inpatient falls.	4.07	0.71
6	4	I think that fall prevention is a high priority in nursing.	4.04	0.59
7	5	I am interested in nursing interventions to prevent falls.	3.99	0.66
8	13	I would feel guilty if a patient fell.	3.80	0.80
9	3	I think that falls in hospitals are an important responsibility for nurses.	3.78	0.67
10	2	I think that inpatient falls are inevitable.^a^	3.55	0.90
11	12	I think that the hospital environment is safe with respect to falls.^a^	3.41	0.85
12	8	I think that fall-prevention education provided upon admission is sufficient.^a^	2.71	0.89
13	9	I think that falls occur because of the patient’s condition.^a^	2.65	0.86
		Total	3.81	0.34

^a^Reverse scored; *M =* mean; *SD =* standard deviation

**Table 4:** Participants’ engagement in fall-prevention activities and average item scores (*N* = 162)

Rank	Item	Items pertaining to engagement in fall-prevention activities	*M*	*SD*
1	20	I always raise the bed rails when moving a patient on a stretcher cart.	3.82	0.47
2	19	I always engage the lock when transferring patients to wheelchairs.	3.77	0.51
3	6	I always raise the bed rails for elderly people, children, unconscious patients, and very unstable patients.	3.73	0.53
4	11	I educate patients to ensure that they ask for help to prevent falls.	3.68	0.53
5	8	I ensure that unconscious patients, very unstable patients, or surgical patients are moved from the bed with assistance from a nurse or caregiver.	3.60	0.62
6	10	I ensure that patients at risk of falling walk with their caregivers.	3.60	0.60
7	7	I ensure that patients at risk of falling who wake up to go to bathroom are helped off the bed by a nurse or guardian	3.52	0.69
8	9	In cases of abuse of drugs that can cause falls, I monitor the occurrence of the drug’s effects.	3.41	0.75
9	1	I inform all inpatients and caregivers of the possibility of falls while introducing them to hospital life.	3.38	0.75
10	13	I educate patients and caregivers in moving to the bed, chair, bathroom, and wheelchair safely.	3.36	0.76
11	16	I ensure that patients wear non-slip shoes of the correct size.	3.31	0.76
12	17	I maintain proper illumination on the bed and in the bathroom.	3.29	0.76
13	15	Paths should be cleared for easy use.	3.28	0.74
14	12	I provide patients and caregivers with instructions on fall prevention and remind them of these frequently.	3.09	0.93
15	14	I encourage high-risk patients to exercise regularly unless it is contraindicated (once per day).	3.01	0.91
16	5	I attach fall hazard signs to patient charts, patient rooms, and beds for high-risk patients.	2.94	1.11
17	4	I assess patients’ levels of normal motor function.	2.93	0.88
18	2	I assess patients’ fall risk factors using a fall risk assessment scale upon admission.	2.91	1.15
19	3	I regularly (e.g., twice) reassess fall risk factors in connection with changes in a patient’s condition after admission.	2.77	1.06
20	18	I place a non-slip mat on the floor when taking a barrel bath or shower.	2.36	1.13

*M =* mean; *SD =* standard deviation

### Relationships between knowledge, attitudes, and engagement in fall-prevention activities

Attitudes about falls were positively related to engagement in fall-prevention activities (*r* = .25, *p* = .001), but no significant relationships were observed between knowledge of falls and engagement in fall-prevention activities (*r* = −.09, *p* = .267) or knowledge of falls and attitudes toward falls (*r* = .09, *p* = .240).

## Discussion

In this study, we investigated nurses’ knowledge of inpatient falls, their attitudes toward inpatient falls, and their engagement in fall-prevention activities in small- and medium-sized hospitals in South Korea. The results of this study showed a mean score of 48.9 for the knowledge of falls, which is lower than the score of 6.84 out of 14 items reported in a previous study [27, 28] measuring knowledge regarding falls among university hospital nurses and a score of 66 reported in another study of nurses in general hospitals [29]. These findings suggest that nurses at small- and medium-sized hospitals’ knowledge regarding falls is lower than that of nurses at tertiary care hospitals. Perhaps this is because small- and medium-sized hospitals offer fewer educational opportunities compared to tertiary care hospitals, which provide consistent patient safety education. The item with the lowest knowledge score regarding falls pertained to consciousness at the time of a fall, followed by those pertaining to frequent diseases that cause falls and bodily injury resulting from falls; these findings were similar to those of previous studies [28]. Fall-prevention methods were the most frequently reported educational content that nurses in the study experienced, whereas the causes and effects of falls were considered to a lesser extent.

Levels of knowledge regarding falls in participants who had experience of patients falling were significantly higher relative to those in participants who had no experience of patients falling. This finding is consistent with the results of a previous study [23, 27] which demonstrated that participants’ knowledge and interest with regard to falls increased once they had experienced patients’ falling.

Mean score for the positivity of nurses’ attitudes regarding falls was higher relative to the scores reported in previous studies using the same questionnaire [20, 27, 29]. The positivity of nurses’ attitudes regarding falls could have increased as the importance of patient safety increased because of small- and medium-sized hospitals’ voluntary participation in the medical institution accreditation system introduced through an amendment to the medical law in June 2010. Attitudes regarding falls varied depending on the duration of work experience. The positivity of attitudes regarding falls in nurses who had worked for over 5 years was higher than reported by the nurses who had worked for less than 5 years. This finding is consistent with previous research reporting significant differences in attitudes based on length of nursing career [23, 27, 29]. This finding might be the result of the higher level of responsibilities and sense of duty found in experienced nurses. Less experienced nurses typically are focused on general nursing activities rather than those involving patient safety. Additionally, the attitudes of nurses regarding falls improved with an increase in the number educational programs on fall prevention that they attended. Therefore, we assume that regular and recurrent fall-prevention education targeted at small- and medium-sized hospitals would reinforce and improve nurses’ attitudes toward patient falls.

Participants’ mean score for engagement in fall-prevention activities was higher relative to the score of 81.5 previously reported in a study of nurses at a general hospital [29] and the score of 77.3 reported in another study of nurses at small- and medium-sized hospitals [17], but was lower relative to the score of 89.8 reported in a study of nurses in geriatric hospitals [20]. The lowest scores were given to Item 18 (e.g., providing a non-slip mat when patients take a barrel bath or shower) and Item 3 (e.g., reassessing fall risk factors after admission). This was consistent with the findings of a study of nurses in a general hospital [29]. It could have been difficult for nurses to reassess fall risk repeatedly and perform fall-related nursing duties regularly in small- and medium-sized hospitals, which have less staff performing fall-prevention duties and patient assessments, relative to those at tertiary care hospitals. Small- and medium-sized hospitals did not undertake fall prevention activities forcefully, and the use of preventive environmental facilities specifically tailored to these hospitals was lacking. Attitudes regarding falls were significantly positively correlated with engagement in fall-prevention activities; however, we found no relationship between nurses’ knowledge regarding and their attitudes regarding falls or engagement in fall-prevention activities. These findings are consistent with previous research [3, 20, 27]. For example, a multifaceted strategy, including reminders and identification systems, audits and feedback, and further education, has been found to effectively increase fall-prevention activities [30].

The findings described above indicate that consistent and recurrent fall-prevention education tailored to small- and medium-sized hospitals should be implemented to promote engagement in fall-prevention activities. Additionally, the positivity of nurses’ attitudes regarding falls was an important factor affecting behavioral intention [17, 31], and the promotion of changes in attitudes regarding falls via introducing attitude-reinforcement programs that consider nurses’ subjective norms must enhance their sense of responsibility and duty. Nurses are considered to play a critical role in fall-prevention activities, including assessing risk factors for falls at patients’ bedsides and providing interventions 24 h a day. Therefore, in consideration of these results, small- and medium-sized hospitals should implement consistent and repeated fall-prevention education tailored to their unique circumstances in order to promote engagement in fall-prevention activities.

### Implications for practice

The present study opens the possibility of increasing nurses’ interest in fall-prevention activities in small- and medium-sized hospitals. Patient safety in small- and medium-sized hospitals can be enhanced by creating an atmosphere wherein developing fall-prevention strategies are voluntary and self-directed (for example, developing a nursing practice guideline for preventing inpatient falls), and providing appropriate motivation and rewards.

### Limitations

This study has some limitations that should be noted. First, data were collected from only seven hospitals in a single city in South Korea; therefore, we cannot generalize the results to a larger population, and this study should be repeated with more nurses from different cities. Second, in our study, the nurses might have understood questions differently, as attitudes and engagement in fall prevention activities were evaluated using self-reported data.

## Conclusions

The present study broadens our understanding of the current situation regarding nurses’ knowledge, attitudes, and prevention activities regarding patient falls in small- and medium-sized hospitals. Nurses’ attitudes regarding falls were positively correlated with their engagement in fall-prevention activities, but their knowledge regarding falls was not. This study confirmed that nurses’ attitudes toward patient safety are critical; considering these findings, a multifaceted implementation program for fall prevention should be implemented to increase the positivity of nurses’ attitudes.

## Data Availability

The datasets used and/or analyzed during the current study are available from the corresponding author on reasonable request.

## Abbreviations

M: Mean
SD: Standard deviation
